# Diurnal Fluctuations in Plasma Hydrogen Sulfide of the Mice

**DOI:** 10.3389/fphar.2017.00682

**Published:** 2017-10-06

**Authors:** Sheng Jin, Bo Tan, Xu Teng, Ruoni Meng, Xin Jiao, Danyang Tian, Lin Xiao, Hongmei Xue, Qi Guo, Xiaocui Duan, Yuming Wu

**Affiliations:** ^1^Department of Physiology, Hebei Medical University, Shijiazhuang, China; ^2^Clinical Pharmacokinetic Laboratory, Shuguang Hospital Affiliated to Shanghai University of Traditional Chinese Medicine, Shanghai, China; ^3^Collaborative Innovation Center for Cardio-Cerebrovascular Disease, Hebei Medical University, Shijiazhuang, China; ^4^Key Laboratory of Vascular Medicine of Hebei Province, Hebei Medical University, Shijiazhuang, China

**Keywords:** hydrogen sulfide, diurnal fluctuations, hydrogen peroxide, 3-mercaptopyruvate sulfurtransferase, oxidative stress

## Abstract

Circadian rhythms are essential in a myriad of physiological processes to maintain homeostasis, especially the redox homeostasis. However, little is known about whether plasma H_2_S exhibits the physiological diurnal variation. The present study was performed to investigate the diurnal fluctuations of plasma H_2_S and explore the potential mechanisms. We found that the plasma H_2_S of the C57BL/6J mice was significantly higher at 19 o’clock than those at 7 o’clock which was not affected by the blood-collecting sequence and the concentrations of plasma cysteine (a precursor of H_2_S). No significant differences in mRNA or protein expression of the CSE, CBS, or MPST were observed between 7: 00 and 19: 00. There were also no significant differences in the CSE and CBS activities, while the activities of MPST in tissues were significantly higher at 19 o’clock. After treatment with AOAA (a CBS inhibitor) or PPG (a CSE inhibitor) for 14 days, plasma H_2_S concentrations at 19 o’clock were still significantly higher than those at 7 o’clock, although they were both significantly decreased as compared with controls. Identical findings were also observed in CSE KO mice. We also found the plasma H_2_O_2_ concentrations were significantly higher at 19 o’clock than those at 7 o’clock. However, H_2_O_2_ concentrations were significantly decreased at 19 o’clock than those at 7 o’clock when mice were exposed to continuous light for 24 h. Meanwhile, the diurnal fluctuations of plasma H_2_S levels and MPST activities in tissues were disappeared. After treatment with DTT for 14 days, there was no significant difference in plasma H_2_O_2_ concentrations between 7 o’clock and 19 o’clock. Meanwhile, the diurnal fluctuations of plasma H_2_S levels and MPST activities in tissues were disappeared. Identical findings were also observed in SOD^2+/-^ mice. When heart tissues were incubated with increasing concentrations of H_2_O_2_ *in vitro*, H_2_O_2_ could dose-dependently increase the activity of MPST within a certain concentration range. In conclusion, our studies revealed that plasma H_2_S concentration and tissue MPST activity exhibited diurnal fluctuations. Modulated by plasma H_2_O_2_ concentration, changes of MPST activity probably led to the diurnal fluctuations of plasma H_2_S.

## Introduction

Circadian rhythms are the natural patterns of physiological, mental, and behavioral changes in living organisms in response to the normal environmental challenges they face over a 24-h period (Panda, 2016). Although circadian rhythms are widespread throughout the body, the circadian timing system is hierarchical. In mammals, the suprachiasmatic nucleus houses the master circadian clock and coordinates a set of peripheral oscillators in different tissues. Molecular regulation by the clock genes in the circadian oscillator is based on interconnected transcriptional- translational feedback loops, whereby feedback to negatively regulate their own transcription (Robles et al., 2017). Moreover, clock proteins are regulated by mechanisms including microRNA, post-translational modification, and proteasomal degradation (Chen et al., 2014; DeBruyne et al., 2015; Hirano et al., 2016). In addition, a growing collection of data suggest that the intracellular redox status play a significant role in regulation of the circadian rhythms, while most redox couples have been reported to undergo circadian rhythms (Pekovic-Vaughan et al., 2014; Schmalen et al., 2014).

As a toxic weak reductant, H_2_S has been accepted as the third “gasotransmitter” after nitric oxide (NO) and carbon monoxide (CO) (Yang et al., 2008). Similar to NO and CO, H_2_S is endogenously generated by several enzymes in mammalian, including CBS, CSE, and MPST. CSE is primarily involved in maintaining cardiovascular function, whereas CBS has an important role in the central and peripheral nervous systems (Abe and Kimura, 1996; Xu et al., 2014). MPST contributes to H_2_S formation in both the brain and the cardiovascular systems (Shibuya et al., 2009; Kimura et al., 2015). It has been demonstrated that H_2_S influences a wide range of physiological processes, including blood vessel relaxation, cardioprotection, neurotransmission, neuroprotection, and insulin secretion (Kimura, 2014). Exogenous H_2_S may also regulate the oxidative stress observed in several diseases sometimes associated with the changes of endogenous H_2_S concentration (Kimura and Kimura, 2004). Recently, Shang et al. (2012) have shown that H_2_S can affect the intracellular redox state via affecting the expression of circadian clock genes mediated by NAD-dependent deacetylase sirtuin-1. Another study finds that cecal H_2_S production by gut microbes from high fat-fed mice exhibit diurnal patterns, which is absent in control mice. Conversely, fecal pellets from high fat-fed mice show loss of rhythmicity in H_2_S production, yet the rhythmicity become evident in control mice (Leone et al., 2015). However, it is not yet clear whether H_2_S in plasma exhibits the physiological diurnal fluctuations.

With this in mind, the aim of present study was to investigate the diurnal fluctuations of plasma H_2_S and explore the potential mechanisms.

## Materials and Methods

### Drugs and Chemicals

Monobromobimane, H_2_O_2_, DTT, AOAA, PPG, *L*-Cysteine, α-ketoglutarate, and pyridoxal-5′-phosphate were purchased from Sigma-Aldrich, Co., Ltd (St. Louis, MO, United States). Detection kit for H_2_O_2_ was purchased from Jiancheng BioEngineering (Nanjing, China). Bicinchoninic acid (BCA) reagent was purchased from Generay Biotechnology (Shanghai, China). Other chemicals and reagents were of analytical grade.

### Animals and Treatments

Male C57BL/6J mice were purchased from Vital River Laboratories (Beijing, China) and acclimatized for at least 2 weeks before experiments. The CSE and superoxide dismutase 2 (SOD2) heterozygote mice with C57BL/6J genetic bases were kindly provided as gifts by Professor Yichun Zhu (Fudan University, Shanghai, China). CSE WT and knockout (CSE KO) mice were used in the experiments while heterozygote mice were maintained for breeding. SOD2 homozygous mice died within 21 days after birth, so heterozygote (SOD2^+/-^) mice were used for the experiments. Mice were housed in plastic cages in a room with a controlled humidity of 60%, at temperature of 22–24°C and on a regular 12-h light and dark cycle (lights on from 7:00 to 19:00). They were fed on standard rat chow and tap water *ad libitum*. At the time of experiments, all mice were 6–8 weeks old. After 2 weeks of acclimatization, the mice were treated as follows:

Male C57BL/6J mice were randomly divided into three groups. Group 1: blood was collected successively at 7:00 and 19:00 from the same mice after anaesthetized with isoflurane (1%); Group 2: blood was collected successively at 19:00 and 7:00 from the same mice; Group 3: blood was collected successively at 7:00 and 19:00 from the same mice which were exposed to continuous light for 24 h. After blood was drawn, the mice in group 1 or 3 were further divided into two subgroups: mice were euthanized with 6% chloral hydrate and tissues (heart, kidney, and liver) were rapidly removed at 7:00 and 19:00, respectively and retained at -80°C until further analysis.

In order to inhibit the activity of CSE, CSE KO mice were used for the experiments. The inhibitors of CSE and CBS, PPG and AOAA, were also used for long-term in C57BL/6J mice as literature (Tan et al., 2017). C57BL/6J mice were injected intraperitoneally with equal volumes of saline, PPG (33.9 mg/kg), or AOAA (17 mg/kg), respectively for 14 days. After treatment, blood was collected successively at 7:00 and 19:00 from the same mice.

In order to observe the effect of ROS on the generation of H_2_S, SOD2^+/-^ mice were used for the experiments. DTT, a thiol-reducing reagent, was also used as a reducer to clean ROS (Deepmala et al., 2013). C57BL/6J mice were injected intraperitoneally with equal volumes of saline or DTT (15.4 mg/kg) respectively for 14 days. After treatment, blood was collected successively at 7:00 and 19:00 from the same mice. SOD2^+/-^ mice or DTT-treatment mice were further divided into two subgroups: mice were euthanized with 6% chloral hydrate and tissues (heart, kidney, and liver) were rapidly removed at 7:00 and 19:00, respectively and retained at -80°C until further analysis.

Plasma was separated from the blood after centrifugation at 3500 rpm for 10 min, and then stored at -80°C until assay.

All our animal experimental procedures were performed according to the Guide for the Care and Use of Laboratory Animals of the National Institutes of Health (NIH) of the United States and approved by the Ethics Committee for Laboratory Animals Care and Use of Hebei Medical University.

### Measurement of H_2_S Concentration in Plasma

The H_2_S in plasma was measured according to previously described methods (Tan et al., 2017). Thirty microliters of plasma were mixed with 80 μL MBB and 10 μL 0.1% ammonia with shaking for 1-h at room temperature for derivatization of sulfide. MBB reacts with sulfide to produce sulfide-dibimane (SDB). SDB is more hydrophobic than most physiological thiols and can be separated by gradient elution and analyzed by liquid chromatography-tandem mass spectrometry. The reaction was then terminated with 10 μL 20% formic acid and centrifuged at 15000 *g* for 10 min. The supernatants were stored at -80°C until H_2_S measurements were done. H_2_S concentrations were determined by using a curve generated with sodium sulfide (0–40 μmol/L) standards.

### Measurement of the Activities of MPST, CSE, and CBS

The activities of MPST, CSE, and CBS in tissues (heart, kidney, and liver) were measured according to the previously described methods with some modified (Tao et al., 2013). Briefly, heart tissues were homogenized in ice-cold PBS and centrifuged at 12,000 *g* for 20 min at 4°C. The supernatant was immediately used to measure the activities of three enzymes, and proteins in the supernatant were quantified using the BCA reagent. To measure the CSE and CBS activity, the enzyme substrate *L*-cysteine (10 mmol/L) and the cofactor pyridoxal-5′-phosphate (2 mmol/L) were added to the supernatant for an incubation of 0.5 h. To measure the MPST activity, *L*-cysteine (10 mmol/L) and α-ketoglutarate (2 mmol/L) were added to the supernatant for an incubation of 0.5 h. Then H_2_S concentrations in the reaction system were measured and the amount of H_2_S produced per microgram protein per hour was calculated as the activities of three enzymes.

### Measurement of H_2_O_2_ Concentration in Plasma

The H_2_O_2_ concentration in plasma was determined by using commercially assay kits according to the manufacturer’s instructions.

### Measurement of Cysteine Concentration in Plasma

The plasma cysteine concentration was measured following a reported LC–MS/MS method with slight modification (Yuan et al., 2012). In brief, the plasma sample was added into four-folder methanol, mixed and centrifuged at 14,000 *g* for 10 min. Supernatant was collected and volatilized in nitrogen gas condition without heating. The residual was redissolved by HPLC mobile phase before analysis. The quantification experiment was conducted by a Prominence HPLC (Shimadzu, Japan) coupled with an AB Sciex API 5500 triple quadrupole mass spectrometer (Foster City, Canada). The chromatographic separation was performed on an Amide XBridge HPLC column (4.6 mm × 100 mm with a particle size of 3.5 μm). Positive ionization mode for electrospray ionization source was used for detection. Data were collected in selected reaction monitoring mode using transitions of *m/z* 122.1 →*m/z* 59.1 for cysteine.

### Western Blot Analysis

Frozen heart tissues were homogenized with ice-cold RIPA lysis buffer. Proteins were extracted and quantified by the BCA method. Equal amount of protein samples were separated on 10% SDS-PAGE gels and transferred to polyvinylidene fluoride (PVDF) membranes. The membranes were blocked with 5% non-fat milk for 1 h and incubated with primary antibodies that recognized CSE (1:1000, Proteintech Biotechnology, Chicago, IL, United States), CBS (1:1000, Abcam, Cambridge, United Kingdom), and MPST (1:1000, Santa Cruz Biotechnology Company, Santa Cruz, CA, United States) at 4°C overnight. Then the membranes were incubated with horseradish peroxidase-conjugated secondary antibodies for 1 h after washing with TBST. Specific bands were detected with SuperSignal West Pico Chemiluminescent Substrate (Thermo, Scientific-Pierce, Waltham, MA, United States). The band intensity was quantified by Image J software.

### Real-time qPCR

Frozen heart tissues were suspended in Trizol reagent (Invitrogen, Carlsbad, CA, United States), and the total RNA was extracted according to the manufacturer’s instructions. Reverse transcription was performed using a Reverse Transcription Kit (Toyobo, Osaka, Japan). A SYBRGreen RT-PCR Kit from Toyobo was used for quantitative real-time qPCR analysis with the StepOnePLUS Real-time PCR system (Applied Biosystems, Foster City, CA, United States), according to the manufacturer’s instructions. Gene-specific primers were used to detect mice CSE (forward primer: 5′-TGCTGCCACCATTACGATTAC-3′; reverse primer: 5′-CTTCAGTCCAAATTC AGATGCCA-3′), CBS (forward primer: 5′-GGCTTTCAGAGACACCTACCA-3′; reverse primer: 5′-ACTCGGGCATAGAGGATTCA-3′), and MPST (forward primer: 5′-CACTCTGACCCTGCTTTGC-3′; reverse primer: 5′-CCACCTTCTTGGCTA CCTCA-3′). The samples were normalized against endogenous mice β-actin (forward primer: 5′-TGTTACCAACTGGGACGACA-3′; reverse primer: 5′-AAGGAAGGC TGGAAAAGAGC-3′), and fold changes were calculated using the formula 2^-ΔΔC_T_^.

### Reaction between H_2_O_2_ and MPST

In order to determine whether H_2_O_2_ could affect the activity of MPST *in vitro*, increasing concentrations of H_2_O_2_ (50 μL, 0–100 μmol/L) were incubated with 450 μL heart tissue proteins after homogenized with ice-cold PBS for 30 min at 37°C. MPST activity was measured as previously described in Section “Measurement of the Activities of MPST, CSE, and CBS.”

### Statistical Analysis

Results were expressed as mean ± SEM. Statistical analysis was performed using an SPSS software package, version 13.0 (SPSS, Inc., Chicago, IL, United States). Comparisons between two time points from one mouse were made using paired-samples *t*-test. Comparisons between two groups were made using Student’s *t*-test. The results for three or more groups were compared using one-way ANOVA followed by Student-Newman-Keuls *t*-test. *P* < 0.05 was considered statistically significant.

## Results

### Diurnal Fluctuations of Plasma H_2_S in Mice

**FIGURE 1:**
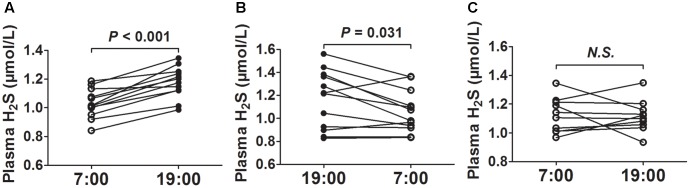
Diurnal fluctuations of plasma H_2_S in mice. **(A)** H_2_S levels in plasma successively at 7:00 and 19:00 from the same mice. **(B)** H_2_S levels in plasma successively at 19:00 and 7:00 from the same mice. **(C)** H_2_S levels in plasma successively at 7:00 and 19:00 from the same mice exposed to continuous light for 24 h. Results are means ± SEM. A *P* of <0.05 was considered significant.

After 2 weeks of acclimatization on a regular 12-h light and dark cycle (lights on from 7:00 to 19:00), we first collected blood at 7 o’clock and then collected blood at 19 o’clock from the same mice. As was shown in **Figure 1A**, the plasma H_2_S concentrations were significantly higher at 19 o’clock than those at 7 o’clock. To rule out the effects of blood-collecting sequence on plasma H_2_S concentrations, we first collected blood at 19 o’clock and then collected blood at 7 o’clock the next day from the same mice. As was shown in **Figure 1B**, the plasma H_2_S concentrations were also significantly higher at 19 o’clock than those at 7 o’clock. So we uniformly collected blood at 7 o’clock and then collected blood at 19 o’clock from the same mice in the subsequent experiment. However, there was no significant difference in plasma H_2_S concentrations between 7 o’clock and 19 o’clock when the mice were exposed to continuous light for 24 h (**Figure 1C**).

### Diurnal Fluctuations of Plasma H_2_S Were Modulated by MPST Activity

**FIGURE 2:**
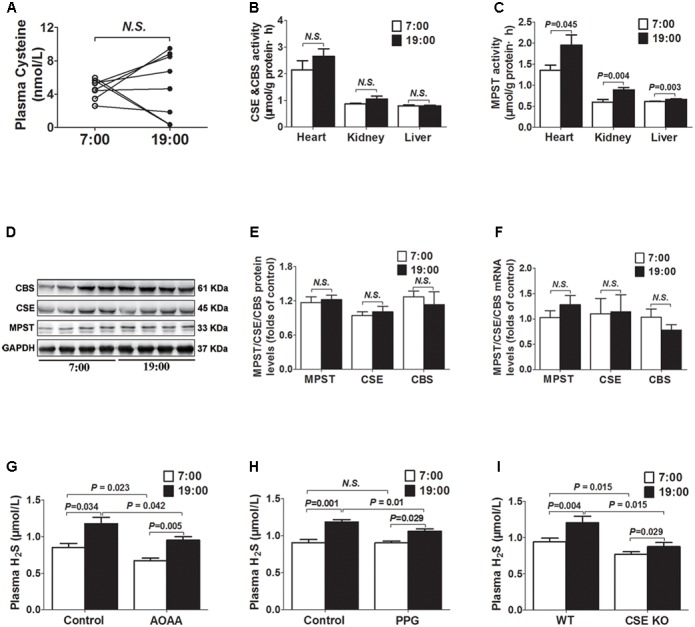
Diurnal fluctuations of plasma H_2_S were modulated by MPST activity. **(A)** Cysteine levels in plasma. **(B)** CSE and CBS activity in tissues. **(C)** MPST activity in tissues. **(D,E)** Representative Western blots and quantitative analysis for CSE, CBS, and MPST protein expression in heart tissues. **(F)** CSE, CBS, and MPST mRNA levels in heart tissues. **(G)** Plasma H_2_S levels in AOAA-treatment mice. **(H)** Plasma H_2_S levels in PPG-treatment mice. **(I)** Plasma H_2_S levels in CSE KO mice. Results are expressed as mean ± SEM. A *P* of <0.05 was considered significant.

As was shown in **Figure 2A**, there was no significant difference in plasma cysteine (the substrate of H_2_S) concentrations between 7 o’clock and 19 o’clock. The activities of MPST in tissues (heart, kidney, and liver) were significantly higher at 19 o’clock than those at 7 o’clock (**Figure 2C**), while neither mRNA or protein expression of the MPST in heart had significant differences (**Figures 2D–F**). There were also no significant differences in activity, protein or mRNA expression of the CSE and CBS (**Figures 2B,D–F**).

After treatment with AOAA (a CBS inhibitor) for 14 days, H_2_S concentrations were significantly decreased either at 7 o’clock or 19 o’clock as compared with control group (**Figure 2G**). However, plasma H_2_S concentrations were still significantly higher at 19 o’clock than those at 7 o’clock. Similarly, after treatment with PPG (a CSE inhibitor) for 14 days, plasma H_2_S concentrations at 19 o’clock were still significantly higher than those at 7 o’clock, although they were significantly decreased at 19 o’clock as compared with controls (**Figure 2H**). In CSE KO mice, H_2_S concentrations were significantly decreased not only at 7 o’clock but also at 19 o’clock as compared with WT mice. However, plasma H_2_S concentrations were still significantly higher at 19 o’clock than those at 7 o’clock in CSE KO mice (**Figure 2I**).

### MPST Activity Was Modulated by Plasma H_2_O_2_

**FIGURE 3:**
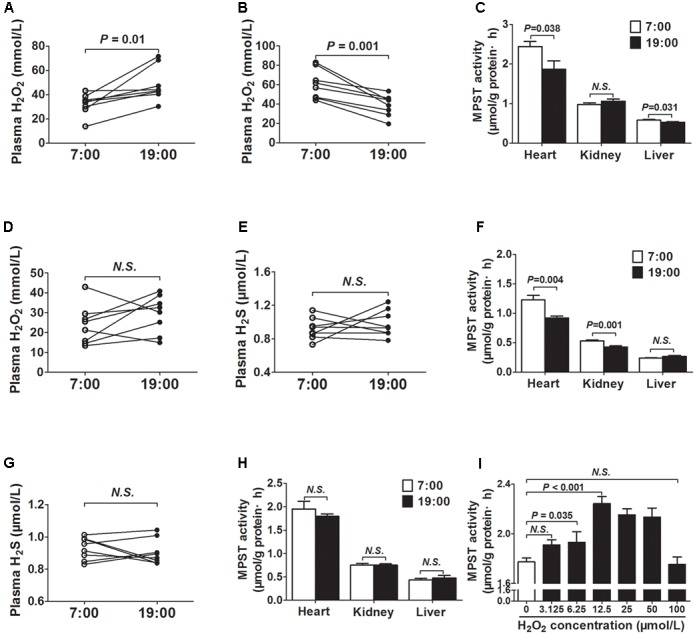
MPST activity was modulated by plasma H_2_O_2_. **(A)** H_2_O_2_ levels in plasma. **(B)** H_2_O_2_ levels in plasma from the same mice exposed to continuous light for 24 h. **(C)** MPST activity in tissues from the same mice exposed to continuous light for 24 h. **(D)** Plasma H_2_O_2_ levels in DTT-treatment mice. **(E)** Plasma H_2_S levels in DTT-treatment mice. **(F)** MPST activity in tissues from the DTT-treatment mice. **(G)** Plasma H_2_S levels in SOD2^+/-^ mice. **(H)** MPST activity in tissues from SOD2^+/-^ mice. **(I)** MPST activity in heart tissues incubated with increasing concentrations of H_2_O_2_ *in vitro*. Results are expressed as mean ± SEM. A *P* of <0.05 was considered significant.

As was shown in **Figure 3A**, the plasma H_2_O_2_ concentrations were significantly higher at 19 o’clock than those at 7 o’clock. To the contrary, H_2_O_2_ concentrations were significantly decreased at 19 o’clock than those at 7 o’clock when mice were exposed to continuous light for 24 h (**Figure 3B**). In addition, MPST activities in tissues (heart and liver) were also decreased at 19 o’clock after 24 h continuous light (**Figure 3C**).

To observe the effect of ROS on the generation of H_2_S, DTT, a thiol-reducing reagent, was also used as a reducer for 14 days to clean ROS. After DTT treatment, there was no significant difference in plasma H_2_O_2_ concentrations between 7 o’clock and 19 o’clock (**Figure 3D**). Meanwhile, the diurnal fluctuations of plasma H_2_S was disappeared (**Figure 3E**). MPST activities in tissues (heart and kidney) were also decreased at 19 o’clock after DTT treatment (**Figure 3F**). In SOD2^+/-^ mice, the diurnal fluctuations of plasma H_2_S was also disappeared (**Figure 3G**). In addition, there was no significant difference in MPST activities of the tissues (heart, kidney, and liver) between 7 o’clock and 19 o’clock (**Figure 3H**).

Next, heart tissues incubated with increasing H_2_O_2_ concentrations were used to determine whether H_2_O_2_ could affect the activity of MPST *in vitro*. As was shown in **Figure 3I**, H_2_O_2_ could increase the activity of MPST within low concentrations.

## Discussion

In the present study, we have focused on the diurnal fluctuations of plasma H_2_S levels and activities of its producing enzymes. There are two important findings: (1) plasma H_2_S concentrations exhibit diurnal fluctuations, which are higher at 19 o’clock than those at 7 o’clock; (2) changes of MPST activity which is modulated by plasma H_2_O_2_ concentrations probably lead to the diurnal fluctuations of plasma H_2_S.

In response to the changes of environment at 24-h rhythm of day and night, most species have evolved endogenous circadian clocks, which regulate the physiological and behavioral activities of the body. Current circadian models are based on transcription/translation feedback loops which clock genes regulate their own transcription and translation over approximately 24 h via a series of interacting negative feedback loops (Robles et al., 2017). In addition, to regulate their own levels of expression, clock genes serve as transcription factors for other genes which regulate a variety of functions, including cell metabolism, immune responses and redox homeostasis (Zhou et al., 2015; Zhu et al., 2017). Many ROS, by products of oxidative stress, antioxidants and enzymes oscillate with circadian rhythmicity (Wilking et al., 2013). Interestingly, we find that the plasma H_2_S, which is a relatively weak reductant and scavenges ROS to alleviate oxidative stress, are significantly higher at 19 o’clock than those at 7 o’clock which is not caused by the blood-collecting sequence in the present study.

The main H_2_S precursor is *L*-cysteine. The other sulfur-containing amino acid, *L*-methionine, is a precursor of *L*-homocysteine, which can be metabolized to cysteines. So, we assess whether the diurnal fluctuations of plasma H_2_S is caused by the differences in *L*-cysteine concentrations. Our results show that there was no significant difference in plasma *L*-cysteine concentrations between 7 o’clock and 19 o’clock. Recently, Tan et al. (2017) report that H_2_S can rapidly and reversibly bind to serum albumin with weaker affinity than that of warfarin. It is similar to many small molecules (such as fatty acids, hormones, and drugs), which are carried by albumin and distributed throughout the body. We also evaluate the concentration of plasma albumin and total proteins at 7 o’clock and 19 o’clock respectively, while there was no significant difference (Supplementary Figures 1, 2).

H_2_S is synthesized endogenously by three enzymes: CSE, CBS, and MPST. The decrease in the activities or expressions of H_2_S-producing enzymes leads to a decrease in H_2_S levels, which is associated with a variety of diseases (Cheng et al., 2016; Greaney et al., 2017). Neither mRNA nor protein of the three H_2_S-producing enzymes is altered at 7 o’clock and 19 o’clock, so we further measure the activities of the three enzymes. Our results show that the activities of MPST in tissues (heart, kidney, and liver) are significantly higher at 19 o’clock than those at 7 o’clock, while there are no significant differences in CSE and CBS. To confirm above results, PPG or AOAA is administrated for 14 days to inhibit the activities of CSE or CBS respectively. After inhibitors treatment, H_2_S concentrations are significantly decreased either at 7 o’clock or 19 o’clock as compared with control group, which is consistent with the previous observation (Roy et al., 2012; Liu et al., 2014). However, plasma H_2_S concentrations are still significantly higher at 19 o’clock than those at 7 o’clock, which is also observed in CSE KO mice. It suggests that the diurnal fluctuations of plasma H_2_S is induced by the changes of MPST activity. Most of the time, the circadian timing system is synchronized by a light stimulus. In photoreceptor cells, the production of H_2_S by MPST is regulated by the levels of its substrate 3-mercaptopyfuvate (3MP), which is produced by cysteine aminotransferase (CAT) whose activity is suppressed by Ca^2+^. In brightness, the voltage-gated Ca^2+^ channels are closed, and the intracellular concentrations of Ca^2+^ in photoreceptor cells are reduced to less than 10 nM, while in darkness, Ca^2+^ ions enter the photoreceptor cells, and the intracellular concentrations of Ca^2+^ reach ∼600 nM. Therefore, the light exposure can regulate H_2_S production by affecting the intracellular concentrations of Ca^2+^ in the retina (Mikami et al., 2011). In order to investigate whether the activity of MPST is regulated by the light, mice are exposed to continuous light for 24 h. We find that the diurnal fluctuations of plasma H_2_S is disappeared and MPST activities in tissues are also decreased at 19 o’clock after 24 h continuous light. It suggests light regulate the activity of MPST which induce the diurnal variation of plasma H_2_S. However, what is the exact mechanism?

Light, the strongest zeitgeber, can generate H_2_O_2_ to regulate the expression of the clock genes and in turn initiate synchronization of rhythms in the zebrafish (Hirayama et al., 2007). However, in the present study, the plasma H_2_O_2_ concentration is significantly higher at 19 o’clock than those at 7 o’clock, while it is significantly decreased at 19 o’clock than those at 7 o’clock when mice were exposed to continuous light for 24 h. The above differences may be due to the different responses to light between nocturnal and diurnal animals. In subsequent experiments, we use DTT, a reductant, to clean ROS. After DTT treatment, there is no significant difference in plasma H_2_O_2_ concentrations between 7 o’clock and 19 o’clock. Meanwhile, the diurnal fluctuations of plasma H_2_S is disappeared. MPST activities in tissues are also decreased at 19 o’clock. In SOD2^+/-^ mice which exhibit persistent oxidative stress conditions (Roos et al., 2013), the diurnal fluctuations of plasma H_2_S and MPST activity are also disappeared. Our results indicate that the diurnal fluctuations of plasma H_2_S and MPST activity are consistent with the diurnal fluctuations of plasma H_2_O_2_. These are congruent with Lin et al. (2013) who find H_2_S production is dependent on NADPH oxidase-derived H_2_O_2_. It has been proposed that a medium concentration H_2_O_2_ can upregulate the expression of the CSE gene in the mammalian cells (Wang et al., 2012). However, there are no significant differences in protein or mRNA expression of the CSE, CBS, or MPST in our studies. Meanwhile, there are also no significant differences activities of CSE and CBS after different treatment (Supplementary Figures 3–5). It suggests that H_2_O_2_ should regulate the activity of MPST. MPST possesses two redox-sensing molecular switches, including a catalytic-site cysteine and an intersubunit disulfide bond, which contributes to redox-dependent regulation of MPST activity (Nagahara, 2013). It has been reported that the redox-sensing molecular switches are easily oxidized by excess molar of specific oxidant, resulting in loss of activity (Nagahara and Katayama, 2005). H_2_O_2_ also can concentration-dependently inhibit H_2_S production in mitochondria isolated from cultured murine hepatoma cells (Módis et al., 2013). In our studies increasing concentrations of H_2_O_2_ are incubated with heart tissue to determine whether H_2_O_2_ can affect the activity of MPST *in vitro*. As shown in the results, low dose of H_2_O_2_ can increase the activity of MPST, while high dose of H_2_O_2_ can inhibit the activity of MPST. These conflicting findings may be due to the different responses of different organs and different cell types. These questions as well as the actual molecular regulation of MPST need to be further investigated.

## Conclusion

Our studies reveal that plasma H_2_S concentration and tissue MPST activity exhibit diurnal fluctuations. Modulated by plasma H_2_O_2_ concentration, changes of MPST activity probably lead to the diurnal fluctuations of plasma H_2_S.

## Author Contributions

SJ, BT, and YW designed and performed experiments, developed methods, analyzed data and contributed to writing the manuscript. XT and DT designed and performed mouse tissue experiments to generate samples for analysis. RM and XJ performed mouse plasma H_2_S analysis. LX and HX performed western blot and qPCR experiments. QG and XD contributed to writing the manuscript.

## Conflict of Interest Statement

The authors declare that the research was conducted in the absence of any commercial or financial relationships that could be construed as a potential conflict of interest.

## Abbreviations

AOAA: amino-oxyacetate
CBS: cystathionine β-synthase
CSE: cystathionine-γ-lyase
CSE KO mice: CSE knockout mice
DTT: dithiothreitol
H_2_O_2_: hydrogen peroxide
H_2_S: hydrogen sulfide
MBB: monobromobimane
MPST: 3-mercaptopyruvate sulfurtransferase
PPG: DL-propargylglycine;
SOD2^+/-^mice: superoxide dismutase 2 heterozygote mice
ROS: reactive oxygen species; WT mice, wild-type mice.
