# Supplementary material for: Diurnal Fluctuations in Plasma Hydrogen Sulfide of the Mice
Source: Front Pharmacol. 2017 Oct 6;8:682. doi: 10.3389/fphar.2017.00682 (PMC5635436; doi:10.3389/fphar.2017.00682)
Supplement: Supplementary file 1 [file Data_Sheet_1.doc]

**Supplementary Figures**

##
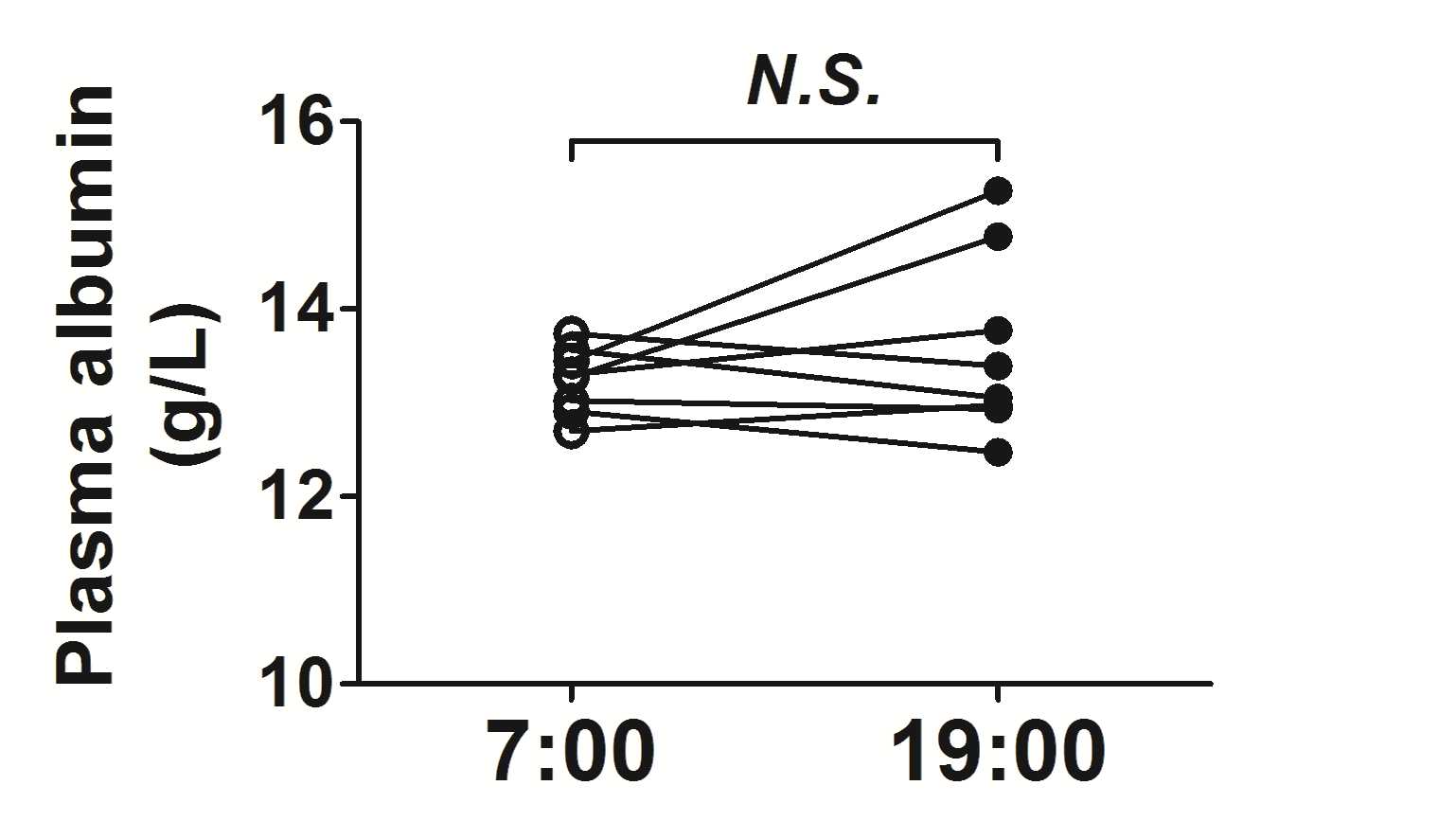


Supplementary Figure 1. Albumin levels in plasma successively at 7:00 and 19:00 from the same mice. Results are means ± SEM. A P of <0.05 was considered significant.


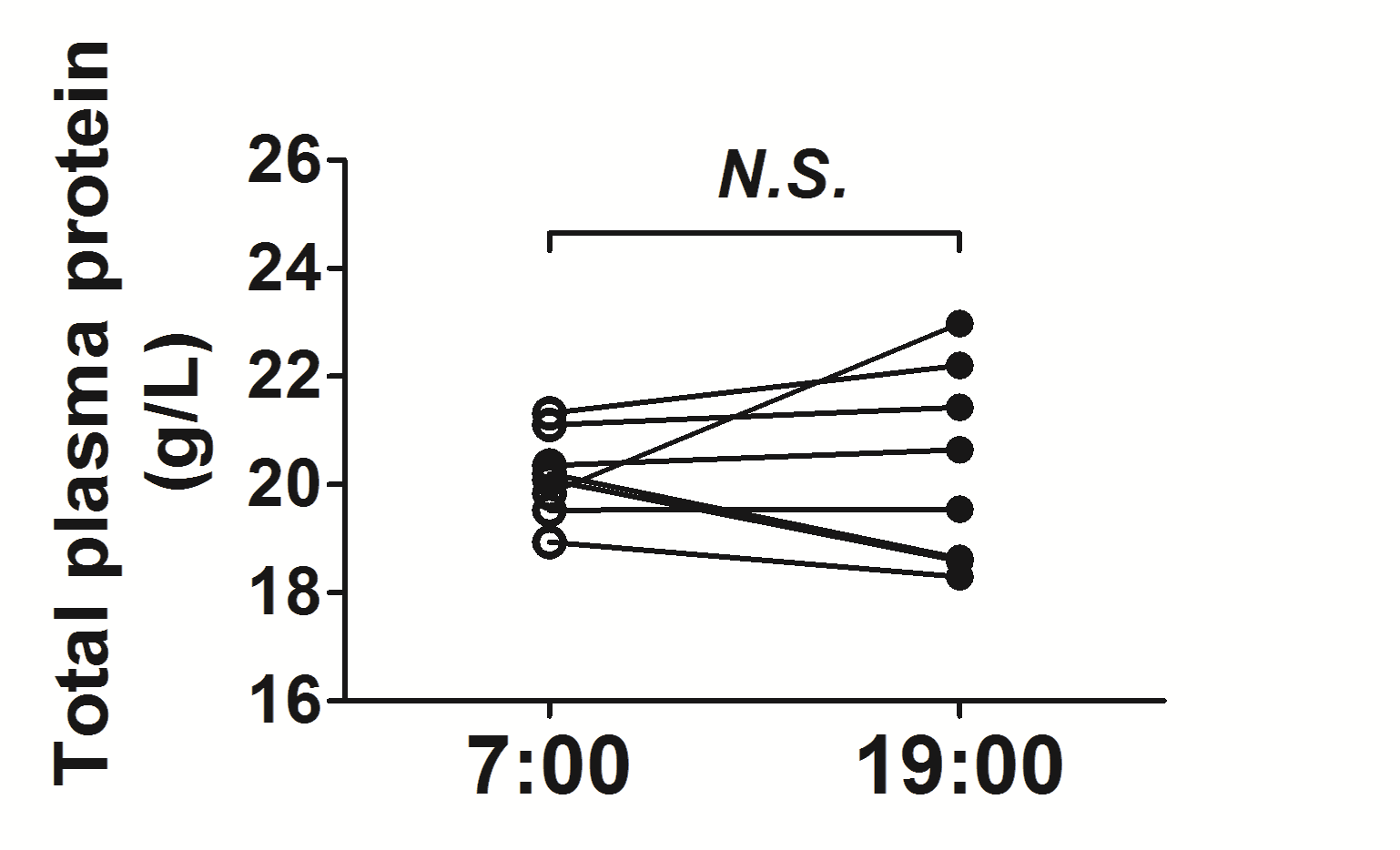


Supplementary Figure 2. Total protein levels in plasma successively at 7:00 and 19:00 from the same mice. Results are means ± SEM. A P of <0.05 was considered significant.


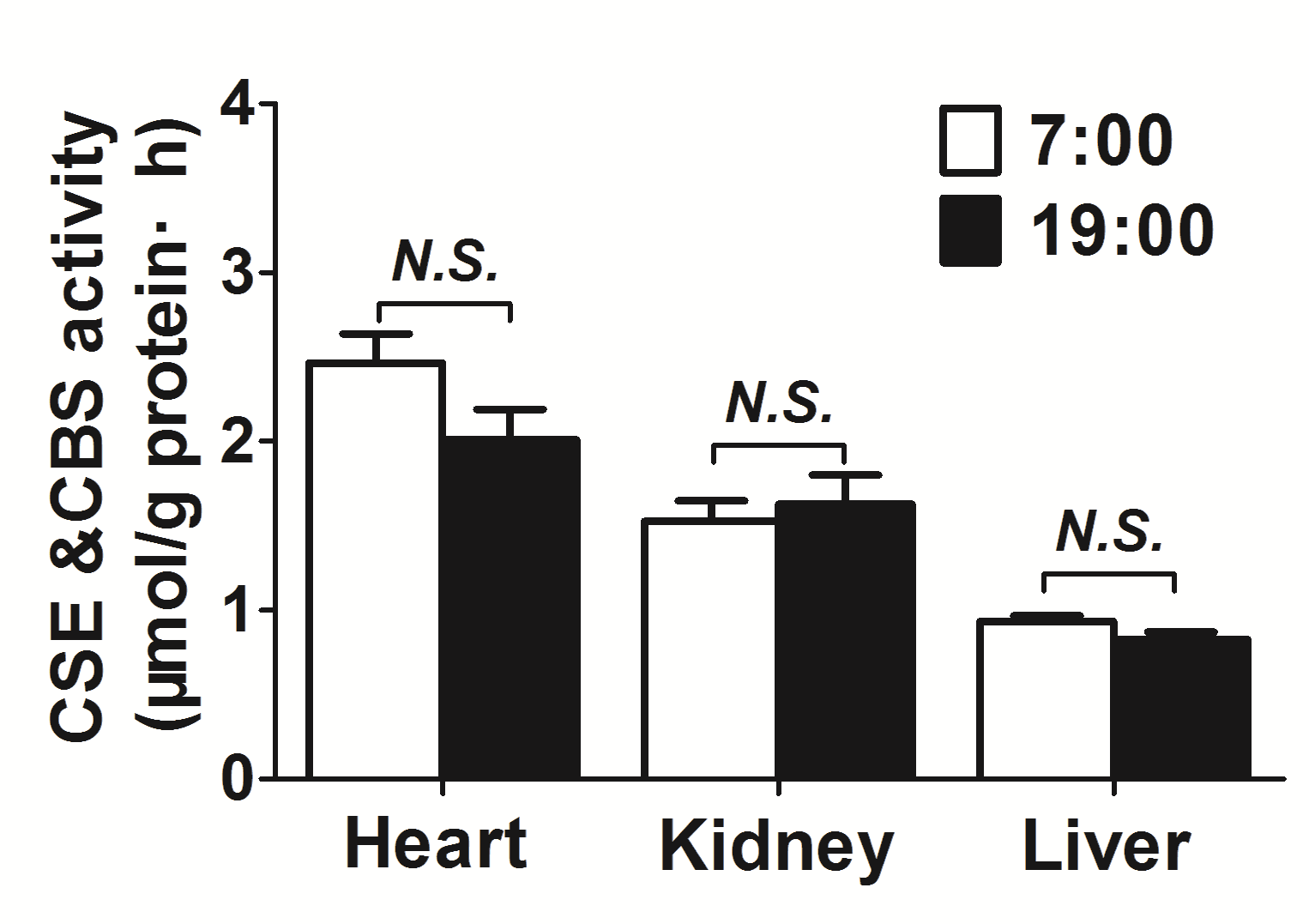


Supplementary Figure 3. CSE and CBS activity in tissues from the same mice exposed to continuous light for 24 hours. Results are means ± SEM. A P of <0.05 was considered significant.


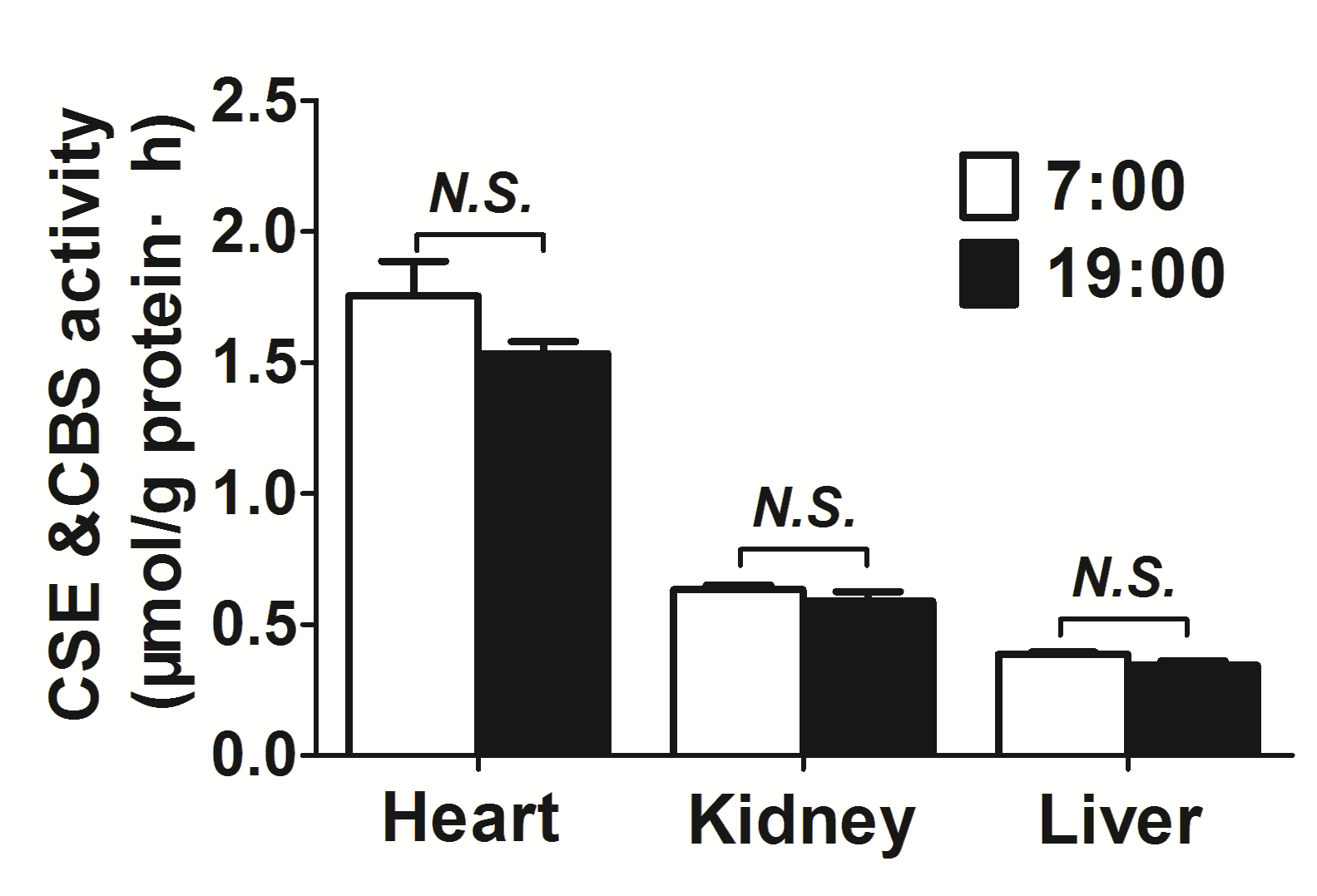


Supplementary Figure 4. CSE and CBS activity in tissues from the DTT-treatment mice. Results are means ± SEM. A P of <0.05 was considered significant.


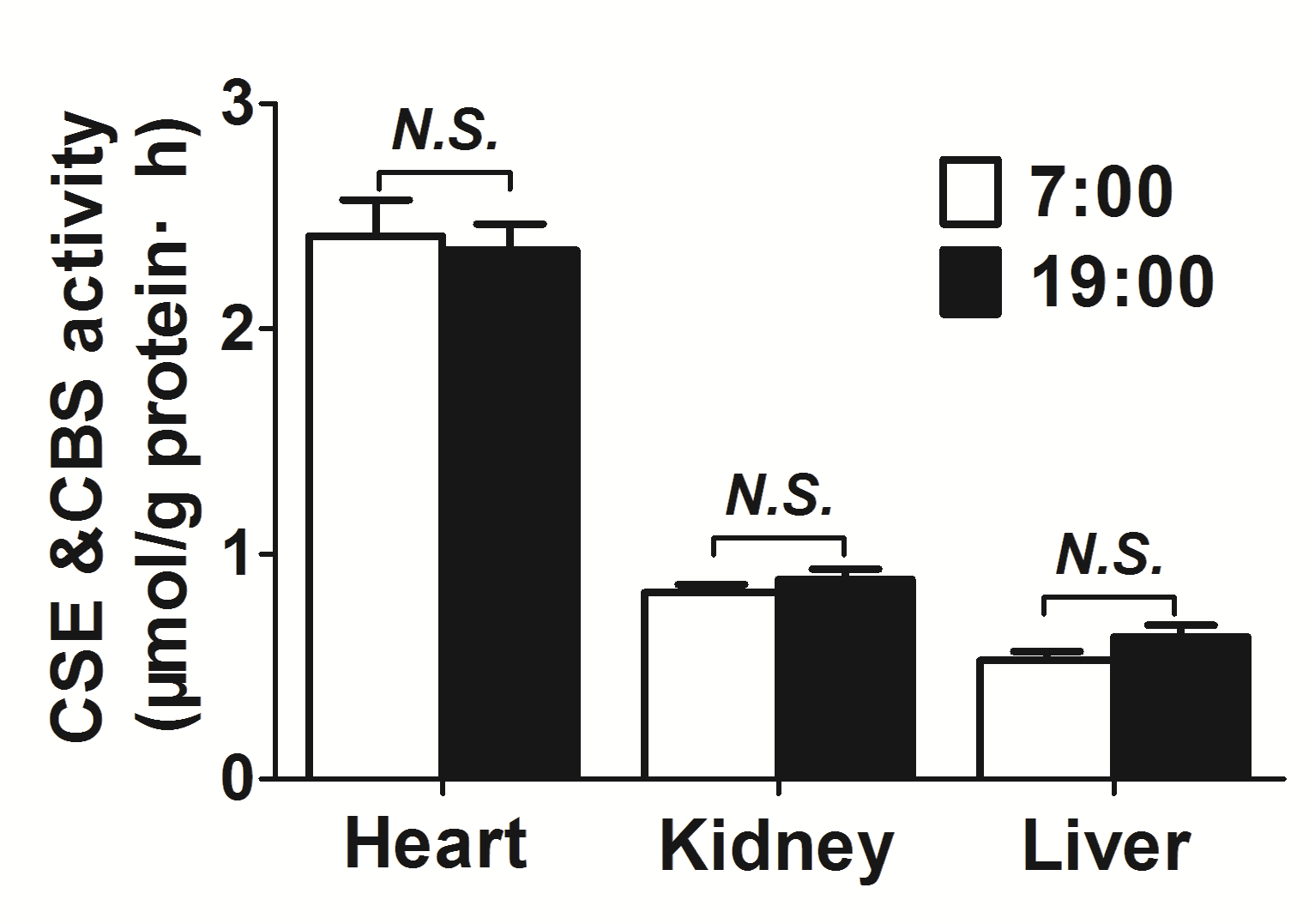


**Supplementary Figure 5.** CSE and CBS activity in tissues from SOD2^+/-^ mice. Results are means ± SEM. A P of <0.05 was considered significant.
